# Predicting Dishonesty When the Stakes Are High: Physiologic Responses During Face-to-Face Interactions Identifies Who Reneges on Promises to Cooperate

**DOI:** 10.3389/fnbeh.2021.787905

**Published:** 2022-02-01

**Authors:** Paul J. Zak, Jorge A. Barraza, Xinbo Hu, Giti Zahedzadeh, John Murray

**Affiliations:** ^1^Center for Neuroeconomics Studies, Claremont Graduate University, Claremont, CA, United States; ^2^Department of Psychology, University of Southern California, Los Angeles, CA, United States; ^3^Association for Computing Machinery, New York, NY, United States

**Keywords:** trust, lie, experiment, economics, cheap talk, deception, cheating

## Abstract

Trust is risky. The mere perception of strategically deceptive behavior that disguises intent or conveys unreliable information can inhibit cooperation. As gregariously social creatures, human beings would have evolved physiologic mechanisms to identify likely defectors in cooperative tasks, though these mechanisms may not cross into conscious awareness. We examined trust and trustworthiness in an ecological valid manner by (i) studying working-age adults, (ii) who make decisions with meaningful stakes, and (iii) permitting participants to discuss their intentions face-to-face prior to making private decisions. In order to identify why people fulfill or renege on their commitments, we measured neurophysiologic responses in blood and with electrodermal activity while participants interacted. Participants (mean age 32) made decisions in a trust game in which they could earn up to $530. Nearly all interactions produced promises to cooperate, although first decision-makers in the trust game reneged on 30.7% of their promises while second decision-makers reneged on 28%. First decision-makers who reneged on a promise had elevated physiologic stress using two measures (the change in adrenocorticotropin hormone and the change in skin conductance levels) during pre-decision communication compared to those who fulfilled their promises and had increased negative affect after their decisions. Neurophysiologic reactivity predicted who would cooperate or defect with 86% accuracy. While self-serving behavior is not rare, those who exhibit it are stressed and unhappy.

## Introduction

People talk. It is quite difficult for individuals in a room not to talk to one another. Yet most studies of strategic decision-making do not allow individuals to talk. When talking is allowed, face-to-face interactions are typically prohibited as biasing decisions (Armstrong, 2006). Face-to-face interactions can influence decisions because of gender, ethnicity, apparel, tattoos, attractiveness, and other aspects that may activate stereotypes (Kurzban et al., 2001; Wright and Sladden, 2003). But the lack of communication severely limits the generalizability of findings to most out-of-lab interactions in which people talk, including business and political negotiations.

Communication can increase understanding of others and reinforce norms of cooperation (Goren and Bornstein, 2000; Chatman and Flynn, 2001), improving outcomes for both parties (Heriyati and Siek, 2011). Pre-decision interactions between parties that are not binding on future decisions, known as “cheap talk,” increase cooperation and decrease defection in money-sharing tasks (Bicchieri, 2002; Bochet et al., 2006; Bicchieri and Lev-On, 2007; Ben-Ner et al., 2011). For example, unrestricted communication in chat rooms increases contributions in public good games more than when only numerical messages are used (Bochet et al., 2006). The positive impact of cheap talk is more likely when it signals behavioral intent (Rousseau, 2001; Bottom et al., 2002) and in settings of incomplete or asymmetric information (Croson et al., 2003). Face-to-face communication may influence behaviors more than remote communication because facial expressions, prosody, and body language combine to indicate one’s intentions (Brosig et al., 2003).

Opportunities to communicate also provide the opportunity to deceive (Hancock et al., 2010). Those who deceive others face censure whereas honoring a promise is perceived as the *status quo* and is unworthy of praise (Gneezy and Epley, 2014). Deception and belief manipulation are key aspects of many strategic interactions, including military operations, bargaining, and poker games (Ettinger and Jehiel, 2010). Yet, concealment and distortion of information require cognitive effort in order to cover up motivations or create events that have not occurred (O’Sullivan et al., 2009). Deception involves several cognitive processes that are metabolically costly including drawing on working memory and inhibiting responses (Johnson et al., 2005; Langleben et al., 2005). Physiologic arousal and/or anxiety during communication is associated with deception (Takahashi, 2005; Olekalns and Smith, 2009). This can be measured by an elevation of stress hormones in blood (Lovallo and Buchanan, 2017) or electrophysiologic measures of arousal such as palmar sweat (Gödert et al., 2001). Unconscious stress responses may therefore signal that someone is untrustworthy. At the same time, it is cognitively demanding for communication partners to monitor the veracity of information and associate it with neurophysiologic cues (Bond and DePaulo, 2006; Forgas and East, 2008; Potts et al., 2019). Most people are unable to consciously detect truth from deception even with training (Vrij and Mann, 2001; Bond and Uysal, 2007; Kraig et al., 2019). It is easier to operate on the assumption of honesty since experience and experiments show that people have a preference to fulfill their promises (Vanberg, 2008).

Group-living creatures such as humans would have had to evolve physiologic mechanisms to identify individuals who are likely to cooperate or defect (Axelrod and Hamilton, 1981; Nowak, 2006). Some of these may be consciously recognizable such as Duchenne smiles (Brown et al., 2003; Mehu et al., 2007) while others are only unconsciously perceived (Zak et al., 2004, 2005a,b; Stellar et al., 2014). Physiologic synchrony can develop during tasks done simultaneously in order to sustain cooperation (Mønster et al., 2016). Yet, the determination of who will cooperate or defect is more difficult when individuals make sequential decisions in private, offering an opportunity to deceive when one’s physiology is unobservable. Choosing to trust another person with the expectation that he or she will later reciprocate, something most people do on a daily basis to a greater or lesser extent, is a type of social dilemma in which assessing future cooperation is essential.

The generalizability of experimental studies of cooperation is limited by the use of convenience samples of college students whose behaviors may not correspond to that in the general population (Henrich et al., 2010). For example, the brain regions that support other-regarding preferences are relatively underdeveloped in adolescents (van den Bos et al., 2011). Money-sharing tasks that seek to measure cooperative behaviors, such as the “trust game” (Berg et al., 1995; Zak et al., 2004, 2005b) are sensitive to framing effects (Burnham et al., 2000; Cronk, 2007), culture (Fershtman and Gneezy, 2001), communication (Buchan et al., 2006), intentions and beliefs (Croson, 2000; McCabe et al., 2003), and show gender differences (Buchan et al., 2008). When college students enter a lab for an experiment, they have knowledge of the cohort with whom they will interact that affects their behavior (Sutter and Kocher, 2007).

Another limitation of most trust experiments is the use of small stakes. Participants are typically endowed with $10 or less, a stake that will not materially affect the lifestyle of most people (Holm and Nystedt, 2008; Camerer, 2011). When stakes are higher, the amount of money sent denoting trust tends to fall (Johansson-Stenman et al., 2005; Johnson and Mislin, 2011). This may indicate a risk-averse preference for a safe payoff. Large stake studies are often conducted in developing countries inducing an additional set of confounds such as existing ethnic or tribal relationships (Henrich et al., 2004; Johnson and Mislin, 2011). The effects of stake size on trust in developed countries is not well-understood because the amount of money at risk may simply not be large enough for patterns to appear.

We sought to address three shortcomings of the extant literature on trust and trustworthiness by studying (i) adults ages 25–50, (ii) who can communicate with one another face-to-face in an unscripted manner prior to (iii) making high-stakes sequential monetary decisions. Participants could earn up to $530 and were encouraged to discuss what they planned to do with their dyadic partner prior to making decisions in private similar to the British TV show Golden Balls (Van den Assem et al., 2012). This design offered the opportunity to measure if promises to cooperate were made and if they were fulfilled or not.

In order to understand the mechanisms that facilitate trust or result in deception, we measured neurophysiologic responses during unconstrained face-to-face communication before participants made decisions in the trust game. We hypothesized that those who reneged on commitments made during face-to-face interactions would have elevated physiologic stress responses compared to participants who fulfilled their promises (Coricelli et al., 2010; Kraig et al., 2019).

## Materials and Methods

### Participants and General Procedure

Seventy-five participants (53% male) provided written informed consent prior to inclusion in this study that was approved by the Institutional Review Boards of Claremont Graduate University (#1006) and the United States Department of the Air Force (#FWR20110168X). Participants were racially diverse, self-identifying as White (38%), Asian (28%), Latino/Hispanic (17%), and African American/Black (17%). Recruitment was limited to working-age adults 25–50 to increase ecological validity (M = 32, SD = 6.94). Recruiting was done on-site at heavy traffic locations (e.g., malls, grocery stores, farmers’ markets) and with an advertisement posted on Craigslist.com. Potential participants were told the purpose of the study was to investigate the physiology of social interactions.

**FIGURE 1:**
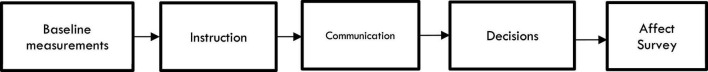
The timeline of the experiment.

Groups of four formed a study cohort with data collected in 2012 at the Center for Neuroeconomics Studies in Claremont, CA, United States. Each cohort participated in two identically structured experimental sessions (Figure 1) lasting approximately 4-h each, 1 week apart, and was randomly formed. Random assignment resulted in DM1-DM2 pairings that were 43.1% mixed gender, 86.3% who fell into different income categories that differed on average by $22,500 per year, and 21.6% who had an age difference of more than 10 years. Since defection is more likely in the final stage of a set of interactions, we report the results of the second session of the study. Measurements were collected in three domains: behavioral (decisions to share money in a trust game), physiological (hormones and electrodermal activity), and self-report (subjective assessments of trust, emotional states). After consent, 18ml of blood was drawn from an antecubital vein after an intravenous catheter was fitted by a registered nurse to establish basal hormone levels and for subsequent blood draws.

### Trust and Communication

**FIGURE 2:**
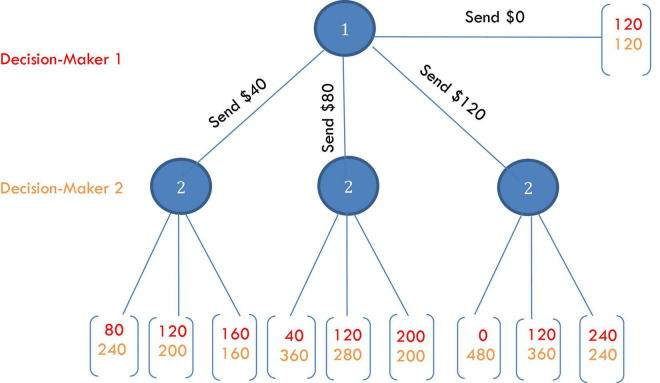
DM1 (red) and DM2 (orange) choices and payoffs in the $120 trust game. Participant earnings in this task varied from $120 to $480.

Participants were fully and identically instructed in a monetary decision task from experimental economics known as the trust game (TG) that was presented using software written by the researchers in Python. The TG matches participants in dyads in which each person is endowed with equal amounts of money and identical information about outcomes is known to both parties. Software randomly assigns participants to the role of Decision-Maker 1 (DM1) or Decision-Maker 2 (DM2). After instruction and examples, DM1 is prompted by software to transfer some amount from his or her endowment to the DM2 in the dyad. Whatever is transferred is removed from DM1’s account and multiplied by a stated value greater than 1 in DM2’s account. The software alerts DM2 of the amount he or she has received from DM1, the total in his or her account and then prompts DM2 to return an amount to DM1. Return transfers from DM2 to DM1 come out of DM2’s account one-to-one and are transferred without multiplication into DM1s account (Figure 2). DM1’s had four options, which were to transfer $0, $40, $80, or $120 to DM2s. In order to have participants engage as both DM1s and DM2s without carry-over effects, DM2s were not informed of the transfer amount. Instead, DM2 was asked to make a decision for each of the amounts received from DM1.

The consensus view in experimental economics is that the DM1 to DM2 transfer measures trust while the DM2 to DM1 transfer captures trustworthiness (Smith, 2010). To ensure that TG instructions were presented correctly and consistently, a set of viewgraphs with voiceover instructions were shown to participants on laptop computers. Neutral language regarding participants was used, for example, avoiding the word “partner” and its synonyms that could signal expected cooperation (Hoffman et al., 2008). In each session, participants were given a quiz before the first TG decision to ensure they understood the consequences of the possible choices. Participants had to pass the quiz before continuing to participate.

Participants were endowed with $120 and made decisions as DM1s and as DM2s in each session. Thus, the total number of observations analyzed from the last session is 150. One of the decisions was selected by die roll for payoff at the end of each session and a randomization algorithm ensured dyads changed across sessions. Participants could earn as little as $50 (the show up amount) or as much as $530. DM2 decisions were made using the strategy method in which decisions were elicited for the four possible DM1 choices without knowing which choice was made. Trust game choices using the strategy method are generally similar to direct decisions (Brandts and Charness, 2011).

Dyadic partners were identified and met each other in advance of decisions. After instruction in the TG, participants were put in a private room inside the lab together for 2 min by themselves to discuss their intentions in the TG. Prior to communicating, participants were encouraged by a research assistant reading a script to make pre-decision commitments to their dyadic partner though they did not have to. Participants were aware that discussions were captured on video for analysis. After the 2-min communication period, participants were led to a lab with partitioned computer stations to make TG decisions. Participants did not receive feedback on how their choices and those of their dyadic partner affecting their earnings in order to eliminate the effects of repeated decisions. After all decisions were made, participants were paid their earnings in cash privately.

This design provides three measures of trust: (i) whether a commitment made was kept or not, (ii) behavior in the TG, and (iii) whether the participant judged the other DM to be trustworthy or not on self-report.

### Stress Hormones

In order to test the hypothesis that during communication promise-breakers will have a stress response, we measured the change in adrenocorticotropic hormone (ACTH) in blood. ACTH is released from the anterior pituitary gland into the blood stream approximately 15 s after an arousing or stressful stimulus and regulates the release of cortisol (Morhenn et al., 2012). ACTH was measured before and after the 2-min communication by drawing blood from an intravenous catheter into an 8ml EDTA-coated whole blood tube using a Vacutainer^®^. Once the blood was drawn, samples were immediately placed on ice and then spun in a refrigerated centrifuge at 4C for 12 min at 1,500 RPM following our previous protocol (Zak et al., 2005b). Blood serum and plasma were separated into 2 ml Fisher brand microtubes and immediately frozen on dry ice. Microtubes were stored in a −80°C freezer until being transferred on dry ice to USC Reproductive Endocrine Research Laboratory for assays. The inter-assay CVs < 12%.

### Electrodermal Procedures

Electrodermal activity (EDA) was recorded as an additional measure of an acute unconscious stress response (El-Sheikh et al., 2008). EDA captures arousal through an increase in conductivity due to sweat from the eccrine glands that are prominent on the hands and feet (Figner and Murphy, 2011; Boucsein, 2012). A BIOPAC MP150 data acquisition system for Windows (Biopac Systems Inc., Goleta, California) was used to measure EDA. Participants were fitted with two disposable Ag–AgCl EDA electrodes on the medial phalanx of the middle and index fingers of the non-dominant hand prior to the start of the experiment. EDA was measured throughout the approximately 4-h session. Baseline EDA was obtained by having participants sit for 2 min at partitioned stations with headphones to mask any background noise.

Electrodermal activity data were cleaned using Acq*Knowledge* software (Biopac Systems Inc., Goleta, California). The data were converted to microSiemens (μS) and square root transformed to correct for positive skew. A semi-automated process was used to correct periods of excessive noise and signal drop that were removed and linearly interpolated as in previous analyses (Johannsen and Zak, 2020). To remove high-frequency noise, a 10-Hz low-pass filter was applied (Norris et al., 2007). All data were visually inspected to ensure that the automatic process accurately identified and corrected artifacts. Following artifact correction, skin conductance levels (SCL, a tonic measure of electrical conductivity) was derived from EDA. The SCL data during the 2-min communication period was used to predict subsequent behavior and was baseline-corrected for each participant.

### Video Recordings

Recordings were made during dyadic communication to assess whether commitments were made. Two independent coders evaluated each interaction as a promise-made (=1), a promise-not-made (=0), or unclear (=9). If a promise to send money was made but the behavioral data were inconsistent with the promise, we categorize this behavior as a promise that was “reneged.” DM2s, who made decisions using the strategy method, reneged if they promised a certain action but failed to follow the promised action. Coders were instructed to identify an interaction as containing a promise if an explicit statement was made by the participant to transfer a specific amount to the interaction partner. Examples include, “I’ll send you all my money when it’s my turn,” and “let’s send everything and equally split.”

Cronbach’s alpha was used to assess intercoder reliability (Cho and Kim, 2015). We took a conservative approach and assigned “unclear” to the “promise-not-made” category. The intercoder reliability was high (reliability coefficient: 0.98; Average inter-item covariance: 0.13).

### Self-Report Measures

Participants completed surveys that assessed personality, attitudes, and mood states. Personality was assessed in order to control for possible confounds. Personality measures included Interpersonal Reactivity Index (IRI; Davis, 1983) to assess trait empathy, trust in others from the World Values Survey (Johnson and Mislin, 2012), prosocial and individualistic preferences were measured using the Social Values Orientation survey (SVO; Declerck and Bogaert, 2008), risk tolerance was assessed using a validated instrument (Weber et al., 2002), and personality traits came from the revised NEO (“big five”) personality inventory (NEO-PI-R; Costa and McCrae, 1992). State affect was assessed at baseline and immediately after decisions using the Positive Affect Negative Affect Schedule that has 20 questions rated using a 1–5 scale (PANAS; Watson et al., 1988).

### Statistical Analysis

Initial analyses tested mean differences and correlations using Student’s *t*-tests. Physiologic predictors of promise-breaking were established using logistic regressions that included changes in physiologic measures during the communication period (ACTH, SCL) and their interaction when appropriate as independent variables. Age, gender, and income were included as controls.

### Data Availability

The data can be downloaded from Open ICPSR, openicpsr-151121.

## Results

### Behavior

**FIGURE 3:**
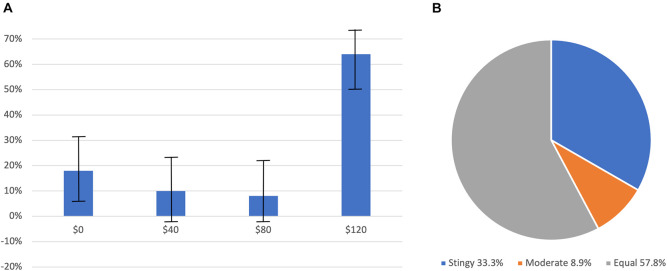
The frequency of choices by Decision-Maker 1s **(A)** and Decision-Maker 2s **(B)**. Error bars in **(A)** are standard errors. **(B)** is the proportion of responses across each non-zero transfer received from DM1.

Nearly two-thirds (64%) of DM1s sent their entire endowments, $120, to the DM2 in their dyad. Nineteen percent of DM1s sent nothing at all, and 17% sent either $40 or $80 (Figure 3). More than two-thirds of DM2s returned half to the DM1s in their dyads. Twenty-one percent of DM2s returned little or nothing, and 8% returned a small share (Figure 3). There was a positive correlation between the money sent by DM1s and the money returned by DM2s (*r* = 0.376, *p* = 0.01). DM1 average earnings were $182.31 while DM2 average was $268.46.

### Commitments

Verbal commitments to cooperate were made by 74 of 75 participants (98.7%) during DM1-to-DM2 and DM2-to-DM1 communications proceeding decisions. Twenty-three DM1s (30.7%) reneged on their commitments, while 21 DM2s (28%) reneged. We will identify DM1s who reneged on a commitment as DM1-Rs and those who kept their commitments as DM1-Cs. Correspondingly, DM2s who reneged on their commitments will be identified as DM2-Rs, and the others as DM2-Cs. The average amount sent by DM1-Rs was 77% less than that sent by DM1-Cs (DM1-R: *M* = $26.09, *SD* = $39.28; DM1-C *M* = $113.85, *SD* = $18.38; df = 26.36; *p* < 0.01). Similarly, DM2-Rs returned 87% less than DM2-Cs (DM2-R: *M* = $29.33, *SD* = $51.20; DM2-C *M* = $222.70, *SD* = $45.41; df = 23.57; *p* < 0.01). Among those who reneged on promises, 13 participants (17.3%) reneged both as DM1 and DM2.

### Electrodermal Activity

**FIGURE 4:**
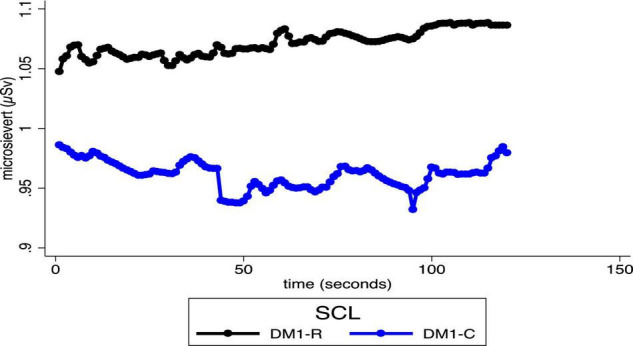
Average skin conductance levels were 12% higher (*p* = 0.03) during the communication period for DM1s who subsequently reneged on their commitment (DM1-R) compared to DM1s who fulfilled their commitment (DM1-C).

Baseline average SCL was not different between DM1-R and DM1-C or DM2-R and DM2-C (*ps* > 0.27). However, during the 2-min communication period, the average baseline-corrected SCL of DM1-Rs was significantly higher than DM1-Cs (DM1-R *M* = 1.07 DM1-C *M* = 0.96; *p* = 0.03) (Figure 4). Average baseline-corrected SCL was not different when comparing DM2-R and DM2-C while they communicated with their dyadic partners (DM2-R *M* = 0.20 DM2-C *M* = 0.24; df = 15.83 *p* = 0.37).

### Endocrine Responses

Average basal hormone levels were within normal ranges (CORT *M* = 14.06 μg/dL, *SD* = 5.99; ACTH *M* = 12.92 μg/dL, *SD* = 7.63; Stewart and Krone, 2011). Post-interaction average ACTH was 38.2% higher in DM1-Rs compared to DM1-Cs (DM1-R: *M* = 15.31, SD = 0.42; DM1-C: *M* = 11.08; SD = 7.27; df = 21; *p* = 0.05). Average ACTH in DM2-Rs did not differ from DM2-Cs (DM2-R: *M* = 13.73, SD = 8.74; DM2-C: *M* = 12.31, SD = 6.82; df = 17; *p* = 0.30).

### Self-Report Measures

**FIGURE 5:**
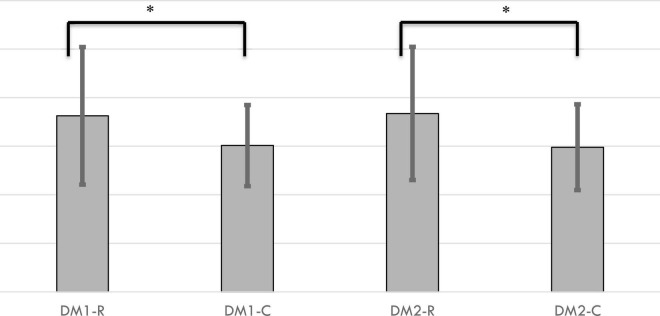
Negative affect was 20% higher for DM1s who reneged on promises (-R) compared to DM1s who fulfilled (-C) promises (*p* = 0.03). Similarly, DM2-Rs had 24% greater negative affect than did DM2-Cs (*p* = 0.02). * = *p* < 0.05.

Both DM1-R and DM2-R reported significantly greater negative affect after their decisions than did DM1-C and DM2-C (DM1-R: *M* = 1.81, SD = 0.71; DM1-C: *M* = 1.51, SD = 0.42; df = 28.99; *p* = 0.03; DM2-R: *M* = 1.84, SD = 0.69; DM2-C: *M* = 1.49, SD = 0.44; df = 25.31; *p* = 0.02; Figure 5). Negative affect did not correlate with ACTH nor SCL in DM1-R or DM2-R (ps > 0.05). Unsurprisingly, DM1-C and DM2-C reported higher trust in other participants (DM1-R: *M* = 3.52, SD = 1.27; DM1-C; *M* = 4.38, SD = 0.69; df = 27.88; *p* = 0.01; DM2-R: *M* = 3.65, SD = 1.04; DM2-C: *M* = 4.37, SD = 0.82; df = 28.50; *p* < 0.01). Neither trait empathy (IRI) nor risk-taking differed between those who reneged on or fulfilled their commitments for both DM1s and DM2s (*ps* > 0.05).

### Promise-Breaker Profile

Age and income (M = $27,289, SD = $21,380) did not vary between DM1 and DM2 participants who kept their commitments and those who reneged (*ps* > 0.05). Women were almost twice more likely to renege as DM1s than were men (Female: 39.4%, Male: 23.8%; *p* = 0.02). DM1-Rs also differed on personality dimensions compared to DM1-Cs. DM1s who reneged on promises to cooperate were less likely to take financial risks (M Renege: 2.76, M Commit: 3.22, *p* = 0.029), were less prosocial (M Renege: 0.59, M Commit: 0.77, *p* = 0.001), and more individualistic (M Renege: 0.40, M Commit: 0.21, *p* = 0.034).

The data showed that DM2-Rs had personality traits similar to DM1-Rs. Compared to DM2s who kept their commitments, DM2-Rs were less prosocial (M Renege: 0.56, M Commit: 0.77, *p* = 0.032), more individualistic (M Renege: 0.43, M Commit: 0.21, *p* = 0.023), had less trait empathy (M Renege: 0.52, M Commit: 0.58, *p* = 0.041), were less likely to take both social risks (M Renege: 4.51, M Commit: 5.65, *p* = 0.0001) and general risks (M Renege: 3.19, M Commit: 3.51, *p* = 0.03), and were less extraverted (M Renege: 0.52, M Commit: 0.58, *p* = 0.018). DM2-Rs did not significantly differ from DM2-Cs by gender (Female: 25.8%, Male: 29.3%; *p* = 0.68). Thirteen promise-breakers reneged on their commitments in both their decisions as DM1s and DM2s. These consistent promise-breakers are a significant proportion of DM1s {*p*[c(1)] = 0.012} and DM2s {*p*[c(1)] = 0.043}.

### Physiologic Predictors of Promise-Breaking

A logit model was estimated to assess if neurophysiologic measures of stress during the communication period could identify DM1s who would renege on commitments to cooperate. The independent variables were ACTH and SCL collected during the communication period and their interaction along with demographic variables (age, gender, and income) as controls. The interaction term was dropped due to a high a variance inflation factor (VIF = 12.49).

The logit model predicted better than chance {*p*[c(1)] = 0.004} which participants would renege with significant variables ACTH (β = −0.21, *p* = 0.05) and gender (β = −3.58, *p* = 0.02). The model classified DM1-Rs and DM1-Cs with 86.2% accuracy, well above the base rate of 69%, with a sensitivity of 71.4% and a specificity of 90.9%

We estimated the same logit model to determine if neurophysiologic variables would predict which DM2s would renege. The ACTH*SCL term did not suffer from multicollinearity and so was included, along with control variables. The model performed poorly {*p*[c(1)] = 0.99} with all physiologic and control variables insignificant (ps > 0.05). Its predictive accuracy only marginally exceed the base rate (model: 74.1%, base rate: 72.0%).

The moderate number of reneging participants may bias a logit analysis (Callas et al., 1998). In order to confirm our findings, we estimated a proportional hazard model using the same variables as above. Both hazard models were well-specified {R^2^: DM1 = 0.081, DM2 = 0.127; Wald test DM1: *p*[c(5)] = 0.004; DM2: *p*[c(5)] < 0.001}. ACTH continued to predict who reneged in both the DM1 and DM2 models with similar hazard ratios (Table 1). In model for DM2, gender was significant and had a very large hazard ratio (3.78) showing for DM2s male gender is a stronger predictor than is stress.

**TABLE 1:** A hazard model confirms that ACTH predicts which DM1s and DM2s will renege on their promises. The model also shows that male DM2s have the highest risk of reneging.

	DM1			DM2		
	
	Coef ± SE	*p*-value	Hazard ratio	Coef ± SE	*p*-value	Hazard ratio
Intercept	−6.04 ± 1.77	0.000		−5.23 ± 1.71	0.002	
ACTH	0.16 ± 0.04	0.000	1.17	0.19 ± 0.04	0.000	1.21
SCL	2.08 ± 0.80	0.009	8.34	0.57 ± 1.28	0.655	1.77
ACTH*SCL		0.198	1.16	0.11 ± 0.12	0.350	1.12
Age	0.05 ± 0.04	0.276	1.07	0.01 ± 0.04	0.803	1.01
Male	0.44 ± 0.58	0.450	1.22	1.32 ± 0.59	0.024	3.78
Income	−0.13 ± 0.17	0.436	0.91	−0.19 ± 0.19	0.318	0.83

## Discussion

Experiments are most valuable when they capture key aspects of out-of-lab environments (Smith, 2003). The present study sought to add a dose of realism to experimental studies of trust and trustworthiness by measuring the behavior of adult participants who could communicate with each other about choices for which the outcomes were financially meaningful. Trust games with all three aspects have not, to our knowledge, been analyzed. Our first finding was that behavior was different. Low stakes trust games in the United States and Europe have average DM1 transfers near 50% of the endowment (Johnson and Mislin, 2011) while in our high stakes protocol transfers in the known last round were substantially higher, 72.4% of the $120 endowment because of the promises made prior to decisions. We sought to identify why people defect in trust games after they have promised to cooperate by measuring neurophysiologic responses during the participant communication period.

While nearly every conversation between dyadic partners resulted in a commitment to cooperate, approximately one-third of DM1s and DM2s reneged on their promises. Our hypothesis that those who reneged would unconsciously “leak out” their intentions was supported for DM1s using two measures of stress, ACTH and SCL. Estimating logistic regressions and a proportional hazard model demonstrated that stress markers accurately predicted distrust by DM1s. The analysis indicates that DM1s who renege on promises appear to know in advance, at least unconsciously, how they will ultimately act. Violating an explicit promise to follow the implicit social norm of cooperation generates a stress response that manifests psychologically as negative mood. DM2s do not appear to know in advance that they will renege on their promises to cooperate since they lack the stress response during the interaction period. Most studies support DM2 responses as being reactive after observing the DM1 transfer rather than planned (Zak et al., 2004, 2005b; Müller and Schwieren, 2020). Nevertheless, when DM2s renege they still experience negative affect similar to DM1s suggesting they know they are committing a norm violation.

The role of stress in inhibiting cooperation has been found in other settings (Takahashi, 2005; Olekalns and Smith, 2009; Willoughby et al., 2012). For example, acute alcohol consumption induces physiologic stress and negative affect causing a reduction in contributions to a shared pool of resources (Zak et al., 2021). Relatedly, male DM2s who are distrusted by DM1s in a $10 trust game experience a spike in the arousal hormone dihydrotestosterone (DHT) and return little or nothing (Zak et al., 2005a). The DHT response is consistent with our finding that for DM2s male gender is a stronger predictor of who will renege than is stress. DM2s who reneged were, by personality, less empathic and were more self-focused than DM2-Cs, traits that are more common in males (Christov-Moore et al., 2014). Contrary to DM2-Rs, DM1-Rs were predominately female although gender was insignificant in the logistic regression for DM1s most likely because of the variation explained by the physiologic variables. DM1-Rs may have decided not to trust DM2s to avoid the risk of non-reciprocation of a large stake. Indeed, DM1-Rs were risk avoidant, a trait that is more pronounced in women (Pawlowski et al., 2008; Charness and Gneezy, 2012).

In addition to being risk averse, DM1-Rs and DM2-Rs were less prosocial than were promise-keepers, consistent with previous findings (Burks et al., 2003; Ben-Ner and Halldorsson, 2010; Thielmann and Hilbig, 2014). Responses to risk are evinced neurophysiologically as stress (Lo and Repin, 2002; Potts et al., 2019) suggesting a trait-state interaction driving promise-breaking. The effect of stress was likely exacerbated by the high stakes DM1s put at risk. Indeed, the substantial effect of stress on DM1s who reneged demonstrates the value of directly measuring neurophysiology in addition to using self-reports.

DM1-Rs and DM2-Rs both experienced negative affect when breaking promises. Negative mood states involve emotions such as guilt, disgust, and fear (Forgas, 2017) and can diminish motivation (Watson, 2000). Induction of a negative mood is associated with increased risk-aversion (Yuen and Lee, 2003) that may have also interacted with personality traits to increase promise-breaking. The trifecta of personality traits, physiological responses to financial risk, and negative affect all contributed to the decision by a non-trivial proportion of DM1s and DM2s to renege on promises made face-to-face to another person. The consequences of cheap talk in the present study appear to be due to the high stakes and inclusion of working-age adults. At least one-third of participants understood the strategy of reneging on promises during the final rounds and had the personality traits and neurophysiologic responses that led them to do so.

## Conclusion

This study demonstrated that cheap talk affects decisions for most people, even during the last round of strategic choices with high stakes at risk. Neurophysiologic indicators of stress accurately predicted which first decision-makers would renege on promises to share money. Participants who were individualistic and risk-averse were also more likely to renege on promises revealing a subtle trait-state interaction. When DM1s and DM2s reneged, both suffered an increase in negative affect revealing the psychological cost of selfishness.

## Data Availability Statement

The original contributions presented in the study are publicly available. This data can be found here: www.openicpsr.org/openicpsr/, 151121.

## Ethics Statement

The studies involving human participants were reviewed and approved by the Institutional Review Boards of Claremont Graduate University (#1006) and the United States Department of the Air Force (#FWR20110168X). The patients/participants provided their written informed consent to participate in this study.

## Author Contributions

PZ, JB, and JM: study conception and design. PZ and JB: data collection. PZ, JB, XH, and GZ: analysis and interpretation of results and manuscript preparation. JM: funding. All authors reviewed the results and approved the final version of the manuscript.

## Conflict of Interest

The authors declare that the research was conducted in the absence of any commercial or financial relationships that could be construed as a potential conflict of interest.

## Publisher’s Note

All claims expressed in this article are solely those of the authors and do not necessarily represent those of their affiliated organizations, or those of the publisher, the editors and the reviewers. Any product that may be evaluated in this article, or claim that may be made by its manufacturer, is not guaranteed or endorsed by the publisher.
